# Synergistic and antibiofilm potential of *Curcuma aromatica* derived silver nanoparticles in combination with antibiotics against multidrug-resistant pathogens

**DOI:** 10.3389/fchem.2022.1029056

**Published:** 2022-11-09

**Authors:** Madhumita S. Tawre, Aishwarya Shiledar, Surekha K. Satpute, Kedar Ahire, Sougata Ghosh, Karishma Pardesi

**Affiliations:** ^1^ Department of Microbiology, Savitribai Phule Pune University, Pune, Maharashtra, India; ^2^ Department of Zoology, Savitribai Phule Pune University, Pune, Maharashtra, India; ^3^ Department of Microbiology, School of Science, RK University, Rajkot, Gujarat, India

**Keywords:** silver nanoparticles, *Curcuma aromatica*, antibiotics, synergy, multidrug-resistant, biofilms

## Abstract

Hospital acquired infections caused due to ESKAPE pathogens pose a challenge for treatment due to their growing antimicrobial resistance. *Curcuma aromatica* (CA) is traditionally known for its antibacterial, wound healing and anti-inflammatory properties. The present study highlights the biogenic synthesis of silver nanoparticles (CAAgNPs) capped and stabilized by the compounds from CA rhizome extract, also further demonstrating their antibacterial, antibiofilm and synergistic effects against multidrug-resistant (MDR) pathogens. CAAgNPs were synthesized using aqueous rhizome extract of CA (5 mg/ml) and AgNO_3_ (0.8 mM) incubated at 60°C up to 144 h. UV-vis spectroscopy, field emission scanning electron microscopy (FESEM), transmission electron microscopy (TEM), energy dispersive spectroscopy (EDS) and X-ray diffraction (XRD) revealed CAAgNPs with characteristic peak at 430 nm, 13 ± 5 nm size of spherical shape, showing presence of silver and crystalline nature, respectively. Dynamic light scattering (DLS) and zeta potential confirmed their monodispersed nature with average diameter of 77.88 ± 48.60 nm and stability. Fourier transform infrared spectroscopic (FTIR) analysis demonstrated the presence of phenolic -OH and carbonyl groups possibly involved in the reduction and stabilization of CAAgNPs. The minimum inhibitory concentrations (MICs), minimum bactericidal concentrations (MBCs) and minimum biofilm inhibitory concentrations (MBICs) of CAAgNPs against *Pseudomonas aeruginosa*, NCIM 5029 and PAW1, and, *Staphylococcus aureus,* NCIM 5021 and S8 were in range from 8 to 128 μg/ml. Almost 50% disruption of pre-formed biofilms at concentrations 8–1,024 μg/ml was observed. Fluorescence microscopy and FESEM analysis confirmed cell death and disruption of pre-formed biofilms of *P. aeruginosa* PAW1 and *S. aureus* S8. Checkerboard assay demonstrated the synergistic effect of CAAgNPs (0.125–4 μg/ml) in combination with various antibiotics (0.063–1,024 μg/ml) against planktonic and biofilm forms of *P. aeruginosa* PAW1. The study confirms the antibacterial and antibiofilm activity of CAAgNPs alone and in combination with antibiotics against MDR pathogens, thus, reducing the dose as well as toxicity of both. CAAgNPs have the potential to be used in wound dressings and ointments, and to improve the performances of medical devices and surgical implants. *In vivo* toxicity of CAAgNPs however needs to be tested further using mice models.

## 1 Introduction

*Pseudomonas aeruginosa* and *Staphylococcus aureus* are amongst the six pathogens belonging to the “ESKAPE” group (*Enterococcus faecium*, *Staphylococcus aureus*, *Klebsiella pneumoniae*, *Acinetobacter baumannii*, *Pseudomonas aeruginosa*, and *Enterobacter* spp.) that are commonly associated with the hospital acquired nosocomial infections. *P. aeruginosa* and *S. aureus* exhibit multidrug-resistance (MDR) and virulence traits boosting the difficulty level of the treatment. The mounting antibiotic resistance has led the requisite for developing alternative strategies which can act on planktonic as well as biofilm forms of the MDR pathogens (Mulani et al., 2019). Moreover, biofilm formation by MDR pathogens contributes for the tolerance towards antibiotics making the treatment challenging (Gaidhani et al., 2014; Kamble and Pardesi, 2020; Tawre et al., 2021).

Medicinal plants or phytochemicals derived from them have been used as traditional medicines for treating bacterial infections. Plants contain a wide range of phytochemicals namely flavonoids, alkaloids, tannins, and terpenoids as their bioactive constituents which are accountable for their biological activity. Few important constituents from the plant extracts are insoluble in water which limits their usage in clinical practice. These constituents are less absorbed due to their inability to cross the lipid membranes of the cells and are highly sensitive to the acidic pH of the stomach, thereby reducing their bioavailability and efficacy (Gafur et al., 2020).

Other alternative strategies such as nanoparticles (NPs), antimicrobial peptides, phage therapy and photocatalytic therapy have been reported against MDR pathogens (Mulani et al., 2019). Advances in nanotechnology has created a benchmark in the field of biomedicine. The biological methods utilize an ecofriendly approach for the synthesis of NPs, over the physical and chemical methods requiring harsh reaction conditions that generate toxic and hazardous by-product (Altinsoy et al., 2019; Ansar et al., 2020; Mostafavi et al., 2022). Amongst the biogenic sources, synthesis of NPs using medicinal plants is widely explored. The phytochemicals from plants serve as reducing, stabilizing and capping agents for the synthesis of NPs hence offering diverse biomedical applications (Mohanta et al., 2020; Ghosh et al., 2021; Javed et al., 2021).

Amongst NPs, silver nanoparticles (AgNPs) are reconnoitered as potential antimicrobial agents and widely accepted for medical applications such as coating of medical devices and surgical implants, preparation of wound dressings and gels (Rai et al., 2009). Medicinal plant-mediated synthesis of AgNPs has attracted researchers due to their promising antibacterial and antibiofilm properties. NPs therefore offer a promising therapeutic platform for the development of innovative biofilm impeders (Koo et al., 2017; Pardesi et al., 2019; Singh et al., 2021). The factors influencing the biological activity of NPs include size distribution, morphology, surface charge, surface chemistry and capping agents (Cheon et al., 2019; Fahimirad et al., 2019). AgNPs synthesized using extracts of *Foeniculum vulgare* (Talank et al., 2022), *Picea abies* and *Pinus nigra* (Macovei et al., 2022), *Zataria multiflora* (Barabadi et al., 2021), *Punica granatum* (Swilam and Nematallah, 2020), *Lysiloma acapulcensis* (Garibo et al., 2020), *Gardenia resinifera* (Parit et al., 2020), *Brassica oleracea* (Ansar et al., 2020), *Piper betle* (Shah et al., 2019), *Prosopis juliflora* (Arya et al., 2019), *Rumex hastatus* (Rashid et al., 2019), *Galega officinalis* (Manosalva et al., 2019) and *Terminalia mantaly* (Majoumouo et al., 2019) have been known to exhibit enhanced antimicrobial activity against *P. aeruginosa* and *S. aureus*. Biofilm inhibition by AgNPs synthesized using extracts of *Zataria multiflora* (Barabadi et al., 2021), *Piper betle* (Shah et al., 2019) *Rhodiola rosea* (Singh et al., 2018a), and *Cannabis sativa* (Singh et al., 2018b) have been reported in *P. aeruginosa* and *S. aureus*. AgNPs have been reported for their cytotoxicity by inducing oxidative stress caused due to the generation of reactive oxygen species (ROS) and free radicals and finally leading to cell death (Tripathi and Goshisht, 2022). Ag^+^ ions disrupts the mechanism of cell division leading to morphological changes in the cell and sudden death (Woo et al., 2008).

*Curcuma aromatica* (CA), a perennial herb, belonging to the Zingiberaceae family is mostly found in India and China. The germacrone component from the hexane extract of CA has been reported for its antimicrobial activity against Gram-positive bacteria (Revathi and Malathy, 2013). Despite the widespread use of CA in traditional medicine for treating various disorders, only sparse literature has scientifically evaluated and validated its therapeutic efficacy. We have previously reported the anticancer activity of AgNPs synthesized using aqueous rhizome extract of CA (CAAgNPs) (Nadhe et al., 2020). These CAAgNPs were found to be less cytotoxic (IC_50_ > 200 μg/ml) against peripheral blood mononuclear cells (PBMCs) proposing their suitability for biomedical applications. Another study has reported the antimicrobial and antibiofilm potential of AgNPs synthesized using CA rhizome extract (Thomas et al., 2018). These AgNPs were incorporated in polymethyl methacrylate thin films which were used against the cariogenic bacterium *Streptococcus mutans*. Considering the limited data available for synthesis, characterization, and applications of CAAgNPs, we present a detailed information on the synthesis and characterization of CAAgNPs using aqueous rhizome extract of CA. The present study also demonstrates the antibacterial and antibiofilm potential of CAAgNPs against representative ESKAPE pathogens (*P. aeruginosa* and *S. aureus*) which are a major challenge to treat any wound infections. Since CA has been traditionally used to treat wound infections, we proposed that the synthesized AgNPs were stabilized by phytochemicals from CA extract offering a novel strategy for treating wound infections. We further tested the synergistic effect of CAAgNPs in combination with antibiotics belonging to different classes against planktonic and biofilm forms of an extensively drug-resistant (XDR) clinical isolate.

## 2 Materials and methods

### 2.1 Plant material and extract preparation

The dried rhizomes of CA were purchased from the local market based in Pune, Maharashtra, India and identified by a botanist from Botanical Survey of India (Western Regional Centre), Pune, Maharashtra, India (Identification No. 1603220017147). CA rhizomes were washed with distilled water, surface sterilized with 70% ethanol and dried under the shade followed by pulverization into fine powder. Aqueous rhizome extract was prepared by heating 0.5 g% (w/v) of CA powder for 1 h. The extract was filtered using Whatman filter paper no. 1 and the filtrate was stored at 4°C until further use.

### 2.2 Microorganisms used

*Pseudomonas aeruginosa* NCIM 5029 (ATCC 27853), *Staphylococcus aureus* NCIM 5021 (ATCC 25923), *Pseudomonas aeruginosa* PAW1 (clinical wound isolate) and *Staphylococcus aureus* S8 (clinical pus wound isolate) were used for antibacterial studies. *P. aeruginosa* PAW1 was concluded as an extensively drug-resistant (XDR) pathogen, resistant to ≥5 classes of antibiotics recommended by Clinical and Laboratory Standards Institute (CLSI, United States, 2018) except for polymyxin B, gentamicin and netilmicin (Tawre et al., 2021). S8 was referred to as MDR pathogen, resistant to ≥3 antibiotic classes (Kamble and Pardesi, 2020).

### 2.3 Synthesis of *Curcuma aromatica* silver nanoparticles

Silver nitrate salt (AgNO_3_, 99.9%) (SRL, India) was procured and used without any further purification. Aqueous stock solution of silver nitrate (100 mM) was prepared in a stoppered volumetric flask and stored in amber colored bottle. CAAgNPs were synthesized using 0.5 g% (w/v) aqueous rhizome extract of CA with varying concentrations of AgNO_3_ (0.4–1 mM) to optimize the parameters for the synthesis. The mixture was incubated at 50°C and was monitored daily for up to 144 h. Color change from yellow to brown was observed by spectral analysis using UV-vis spectroscopy (Spectra Max M2, Molecular Devices, United States). AgNO_3_ concentration showing maximum peak for the synthesis of CAAgNPs was chosen and further optimization at different temperatures viz, 50, 60, and 70°C for 144 h was done. The maximum peak for the synthesis of CAAgNPs was read using UV-vis spectroscopy. Synthesized CAAgNPs were concentrated in a freeze drier (approximately 10 mg/ml) and stored at 4°C until further use.

### 2.4 Characterization of *Curcuma aromatica* silver nanoparticles

The surface morphology and particle size of CAAgNPs were analyzed using field emission scanning electron microscopy (FESEM) (FEI Nova Nano SEM 450, Netherlands) and transmission electron microscopy (TEM) (Tecnai G^2^ 20U FEI, Netherlands). A drop of CAAgNPs was dried on glass slide and copper grid for FESEM and TEM analysis respectively (Singh et al., 2018b). Thin CAAgNPs films on glass slides were prepared for analysis of phase formation using X-ray diffractometer (XRD) (D8 Advanced Brucker, Germany) with a Cu Kα (1.5 Å) source (Ghosh et al., 2012). The presence of silver ions in the CAAgNPs was detected by energy dispersive spectrometer (EDS) (JED-2300; JEOL) equipped with TEM at an energy range 0–20 keV. The functional groups present in the CAAgNPs were identified using Fourier-transform infrared (FTIR) spectroscopy (Jasco FT/IR-6100, Japan). CAAgNPs powder was mixed with potassium bromide and exposed to an infrared source of 400–4,000 cm^−1^. Similarly, aqueous rhizome extract of CA was concentrated in a freeze drier and the powder was processed for FTIR analysis (Ghosh et al., 2012). The hydrodynamic diameter and zeta potential of the CAAgNPs were measured using dynamic light scattering (DLS) analysis (Nano-ZS90, Malvern, United Kingdom) (Barabadi et al., 2021).

### 2.5 Antibacterial studies

#### 2.5.1 Effect of *Curcuma aromatica* silver nanoparticles on MDR/XDR pathogens

##### 2.5.1.1 Minimum inhibitory concentrations (MICs) and minimum bactericidal concentrations (MBCs)

MICs of synthesized CAAgNPs (10 mg/ml stock solution) were tested against *P. aeruginosa*, NCIM 5029 and PAW1 and, *S. aureus*, NCIM 5021 and S8 using broth microdilution method (CLSI, United States, 2021). Briefly, O.D. adjusted culture having 10^5^ CFU/ml in Luria Bertani (LB) broth was added to the microtiter plate. CAAgNPs were added to the microtiter plate at concentrations ranging from 2 to 1,024 μg/ml by serial dilution. The aqueous rhizome extract of CA (100 mg/ml; concentrated in a freeze drier) was also tested at concentrations ranging from 2 to 4,000 μg/ml. The wells having LB medium alone and culture inoculated LB medium were considered as negative and positive controls respectively. The plates were read at 540 nm using a microplate reader at 0 h and incubated at 37°C to note the readings after 24 h. MBCs were determined by spotting 10 µl of the medium from each well of the microtitre plates on to LB agar plates. The minimum concentration at which there was no growth was considered as MBC. The experiment was performed in triplicates.

#### 2.5.2 Effect of *Curcuma aromatica* silver nanoparticles on biofilms formed by MDR/XDR pathogens

The effect of CAAgNPs on biofilms was evaluated through biofilm inhibition and disruption assays against *P. aeruginosa*, NCIM 5029 and PAW1 and, *S. aureus*, NCIM 5021 and S8. Minimum biofilm inhibitory concentrations (MBICs) of CAAgNPs were determined using crystal violet staining assay (Gaidhani et al., 2014). Briefly, LB containing 10^5^ CFU/ml of the test isolate was added to the wells of the microtiter plate. CAAgNPs were then added at concentrations ranging from 2 to 1,024 μg/ml. Similarly, MBICs of CA rhizome extract were determined at concentrations ranging from 2 to 4,000 μg/ml. The wells having LB medium alone and culture inoculated LB medium were considered as negative and positive controls respectively. Plates were incubated for 24 h at 37°C. After incubation, the bacterial cell suspension was slowly aspirated without disturbing the biofilm to remove the planktonic cells and wells were washed twice with phosphate-buffered saline (PBS). Biofilms were stained with 0.1% crystal violet at 37°C for 10 min. The wells were then washed with PBS and allowed to air dry. The stained biofilms were then solubilized in 33% acetic acid. The biofilm formation was measured at 590 nm using a microtiter plate reader. The experiment was performed in triplicates.

Similarly, biofilm disruption assay was performed using O.D. adjusted culture as mentioned above. Plates were incubated at 37°C for 24 h to allow the biofilm formation. CAAgNPs were then added to the pre-formed biofilms at varying concentrations (2 to 1,024 μg/ml) and plates were further incubated at 37°C for 24 h. The treated biofilms were measured using the protocol as described above. The experiment was performed in triplicates. Percent biofilm inhibition or disruption was calculated as follows.

$$Percent\;biofilm\;inhibition\;or\;disruption=\frac{\left(A_{590nm}\;without\;CAAgNPs-A_{590nm}\;with\;CAAgNPs\right)}{A_{590nm}\;without\;CAAgNPs}X\;100$$

where A stands for absorbance.

#### 2.5.3 Live/dead staining of *Curcuma aromatica* silver nanoparticles treated pre-formed biofilms of MDR/XDR pathogens

Fluorescence microscopic analysis was performed for the qualitative estimation of the cell viability in the pre-formed biofilms of *P. aeruginosa* PAW1 and *S. aureus* S8 treated with and without CAAgNPs using LIVE/DEAD BacLight™ Bacterial viability kit (Invitrogen, California). Briefly, biofilm was allowed to form on the sterile cover slips for 24 h at 37°C (Tawre et al., 2021). Further, the pre-formed biofilm was treated with CAAgNPs at their respective MBICs for 24 h at 37°C. Untreated biofilm was kept as control. The cover slips were then removed from the medium and washed twice with PBS. The biofilms were stained with BacLight dye mixture and incubated for 15 min under dark conditions. The biofilms were then washed twice with PBS to remove the excess stain. The fluorescence from live (green) and dead (red) cells was observed using filters with excitation wavelength of 450–490 nm and 545–570 nm respectively under fluorescence microscope (Zeiss Axioscope A1, Germany) with 100× objective. The images were processed using ImageJ software.

#### 2.5.4 Field emission scanning electron microscopy of *Curcuma aromatica* silver nanoparticles treated pre-formed biofilms of MDR/XDR pathogens

Effect of CAAgNPs on pre-formed biofilms of *P. aeruginosa* PAW1 and *S. aureus* S8 was observed under FESEM at ×30,000 magnification. Briefly, LB containing 10^5^ CFU/ml of culture was dispensed in 12 well plates. Sterile glass slides of approximately 5 × 5 mm in size were placed in each well. The plates were incubated at 37°C for 24 h and biofilm was allowed to form on the surface of the slide. Further, the pre-formed biofilm was treated with CAAgNPs at their respective MBICs for 8 h at 37°C. Untreated biofilm was kept as control. Glass slides containing biofilm were then removed from the medium, washed twice with PBS and processed for FESEM analysis (Tawre et al., 2021). Briefly, the biofilms were fixed with 2.5% glutaraldehyde at 4°C for overnight and then washed with PBS. Further, biofilms were washed with a series of ethanol gradations 20, 40, 60, 80 and 90% for 15 min each, and twice with absolute ethanol. The slides were air-dried, coated with gold, and observed under FESEM at 30,000× magnification.

#### 2.5.5 Synergistic effect of *Curcuma aromatica* silver nanoparticles in combination with antibiotics against planktonic and biofilm forms of *P. aeruginosa* PAW1

The synergistic effect of CAAgNPs in combination with antibiotics was evaluated using the checkerboard assay using an XDR *P. aeruginosa* PAW1 isolate. Seventeen antibiotics were selected for the synergy assays against *P. aeruginosa* PAW1 as per our earlier report (Tawre et al., 2021). Briefly, O.D. adjusted culture (10^5^ CFU/ml) in LB broth was added to the microtiter plate. Checkerboard assay was performed using each antibiotic (0.0625–1,024 μg/ml) tested in combination with CAAgNPs (0.25–8 μg/ml) (Berenbaum, 1978). Antibiotics alone and CAAgNPs alone were also tested. Antibiotics were serially diluted from higher to lower concentrations and CAAgNPs were added to each well of a row at selected concentration. The wells having LB medium alone and culture inoculated LB medium were considered as negative and positive controls respectively. Plates were incubated at 37°C for 24 h and O.D. was read at 540 nm. The experiment was performed in triplicates. Percent viability for each combination was determined and fractional inhibitory concentration (FIC) index was calculated as follows:

$${FIC}_{A}=\frac{{MICs}_{A}\;in\;combination}{{MICs}_{A}}$$

$${FIC}_{N}=\frac{{MICs}_{N}\;in\;combination}{{MICs}_{N}}$$

$$\sum FIC={FIC}_{A}+{FIC}_{N}$$

where A indicates antibiotics; N indicates CAAgNPs.

The interaction was described as synergistic if FIC index value was ≤0.5, additive if 0.5 < FIC≤ 1, indifferent if 1 < FIC< 2 and antagonistic if FIC ≥2. The effect of the combination on *P. aeruginosa* PAW1 viability was represented in the form of heat plots indicating the interactions as synergistic, additive, or antagonistic (Habash et al., 2017).

Biofilm inhibition assay was performed using checkerboard assay as described above using a combination of CAAgNPs with antibiotics. Crystal violet staining assay was performed as described above and percent biofilm inhibition was determined. The experiment was performed in triplicates.

### 2.6 Statistical analysis

All the experiments were replicated three times for each assay and the results were determined as means ± SD. Statistical analysis using one-way ANOVA was carried out followed by Tukey’s HSD post hoc test for biofilm inhibition and disruption assays. Differences were considered statistically significant at *p* < 0.01 or *p* < 0.05. Graphical analysis of the data was performed using Graph pad prism 9.

## 3 Results

### 3.1 Synthesis and characterization of *Curcuma aromatica* silver nanoparticles

**FIGURE 1:**
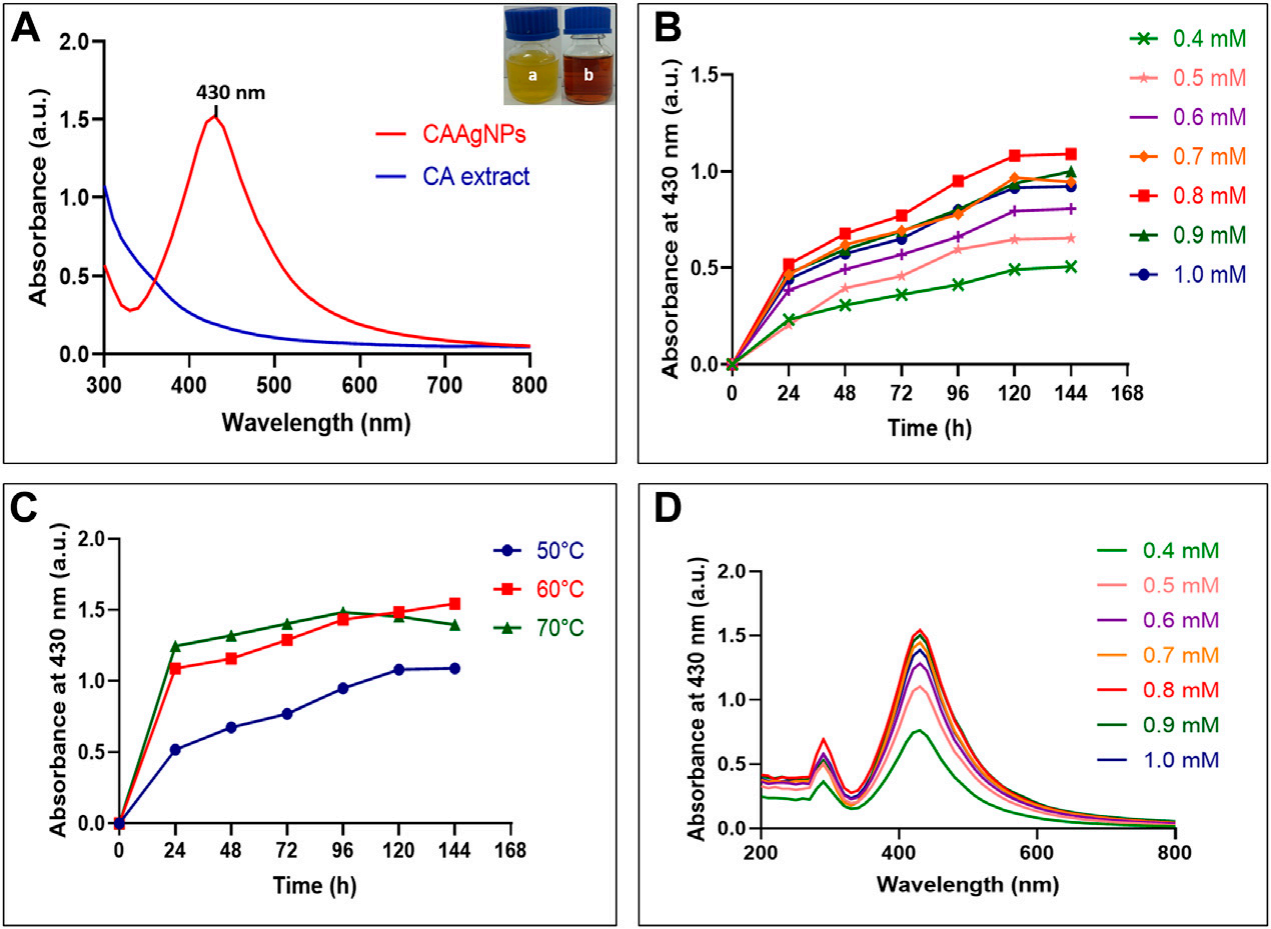
Optimization of CAAgNPs synthesis **(A)** UV-visible spectrum for aqueous CA rhizome extract and synthesized CAAgNPs (maximum peak at 430 nm). “a” in the inset indicates yellow colored aqueous CA rhizome extract before synthesis and “b” indicates brown colored CAAgNPs after synthesis. Time course of CAAgNPs synthesis at **(B)** 0.4, 0.5, 0.6, 0.7, 0.8, 0.9, and 1 mM concentration of AgNO_3_, and **(C)** 50, 60, and 70°C temperatures. **(D)** UV-visible spectrum for CAAgNPs synthesis using range of concentrations of AgNO_3_ at 144 h.

Gradual color change from yellow to brown was observed during CAAgNPs synthesis with a strong surface plasmon resonance (SPR) band within an average of λ_max_ 430 nm (Figure 1A). Optimization study revealed that 0.5 g% (w/v) aqueous rhizome extract of CA and 0.8 mM of AgNO_3_ incubated at 60°C for 144 h gave the maximum synthesis of CAAgNPs (Figures 1B,C). Spectrum analysis displayed the maximum peak of CAAgNPs at 0.8 mM concentration of AgNO_3_ (Figure 1D).

**FIGURE 2:**
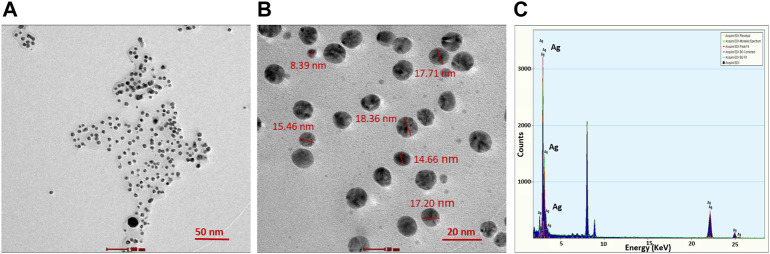
TEM imaging of CAAgNPs at **(A)** 50 nm and **(B)** 20 nm scales revealed the spherical shape and size in the range of 13 ± 5 nm. **(C)** EDS analysis indicated the peak for the presence of Ag^+^ at 3 KeV.

**FIGURE 3:**
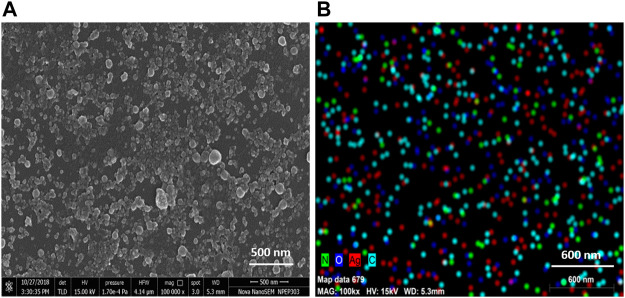
**(A)** SEM imaging of CAAgNPs revealed spherical and uniform shape observed under 1,00,000 × magnification at 500 nm scale. **(B)** Elemental mapping of silver (red color) in CAAgNPs using EDS at 600 nm scale. Uniform distribution of C, O, and Ag elements on the surface of CAAgNPs indicating successful coating.

**FIGURE 4:**
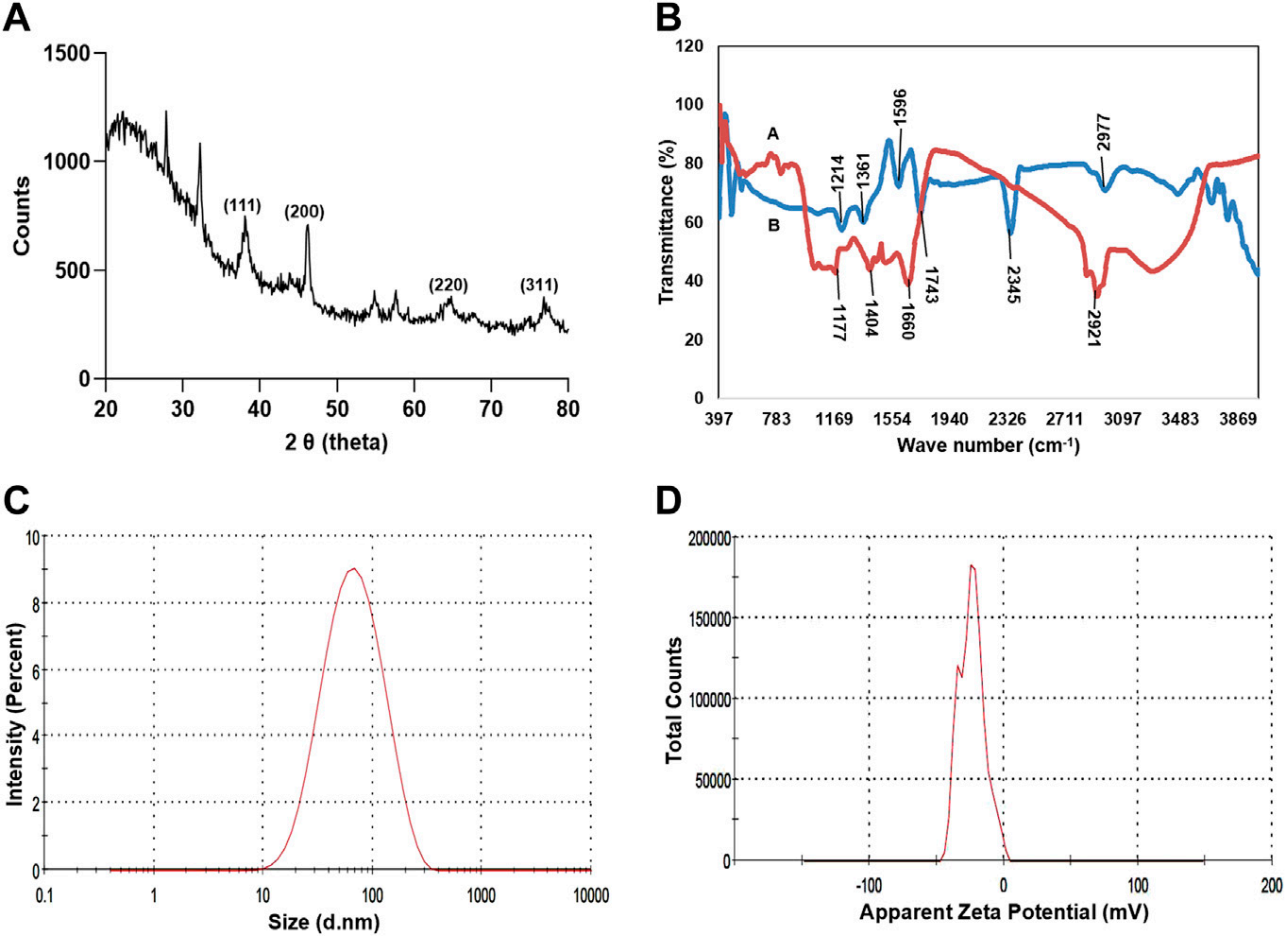
Characterization of CAAgNPs **(A)** XRD analysis showed diffraction peaks at 38°, 44.86°, 64.70°, and 77.4° corresponding to (111), (200), (220) and (311) planes of a face-centered cubic (fcc) structure of silver crystals revealing crystalline nature. **(B)** FTIR analysis of CAAgNPs represented by “A (red line)” indicates a peak at 1,404 cm^−1^ related to the presence of C-C, 1,660 cm^−1^ related to C=O and 2,921 cm^−1^ for the extension of the C-H bond with alkanes vibration and aldehyde C-H stretching. FTIR analysis of CA rhizome extract represented by “B (blue line)” indicates a peak at 1,214 cm^−1^ related to aromatic CO stretching vibration, 1,361 cm^−1^ related the olefin bending vibration of the CC group bound to the benzene ring of the curcumin, 1,596 cm^−1^ related to C=C double-bond stretching, and 2,977 cm^−1^ related to C-H stretching **(C)** DLS analysis confirmed the uniformity and average hydrodynamic diameter of CAAgNPs to be 77.88 ± 48.60 nm. **(D)** Zeta potential at −23.8 mV indicated stability of CAAgNPs.

TEM analysis revealed that the CAAgNPs were monodispersed with spherical shape and 13 ± 5 nm in size analyzed using ImageJ software (Figures 2A,B). The peak at 3 KeV was observed in EDS analysis confirming the presence of elemental silver (Figure 2C). FESEM analysis endorsed spherical morphology and uniformity in the shape of CAAgNPs (Figure 3A). Elemental mapping conducted through EDS demonstrated the presence of silver (Figure 3B). It was also evident that elements of C, O, and N were uniformly distributed on the surface of CAAgNPs suggesting the presence of phytochemicals involved in the stabilization of CAAgNPs. XRD analysis showed diffraction peaks at 38.0°, 44.86°, 64.70°, and 77.4° and these corresponds to (111), (200), (220), and (311) planes of a face-centered cubic (fcc) structure of silver crystals (Figure 4A). The data was matched with the standard Joint Committee for Powder Diffraction Set (card 040783) confirming a face-centered cubic structure for CAAgNPs. Functional groups associated with aqueous rhizome extract of CA and CAAgNPs were identified by FTIR (Figure 4B). The FTIR analysis of aqueous rhizome extract of CA indicated peaks at 1,214 cm^−1^ related to aromatic CO stretching vibration, 1,361 cm^−1^ related the olefin bending vibration of the CC group bound to the benzene ring of the curcumin, 1,596 cm^−1^ related to C=C double-bond stretching, and 2,977 cm^−1^ related to C-H stretching. The FTIR results of CAgNPs indicated peaks at 1,404 cm^−1^ related to the presence of C-C, in which CH_3_ bending occurred; 1,660 cm^−1^ related to C=O with carbonyl stretching and the existence of amide-1 (-NHCO of amide), where the proteins are bent; 2,921 cm^−1^for the extension of the C-H bond with alkanes vibration and aldehyde C-H stretching. DLS illustrated the average hydrodynamic diameter of CAAgNPs to be 77.88 ± 48.60 nm (Figure 4C) and the -23.8 mV of zeta potential (Figure 4D).

### 3.2 Antibacterial and antibiofilm activity of *Curcuma aromatica* silver nanoparticles

**FIGURE 5:**
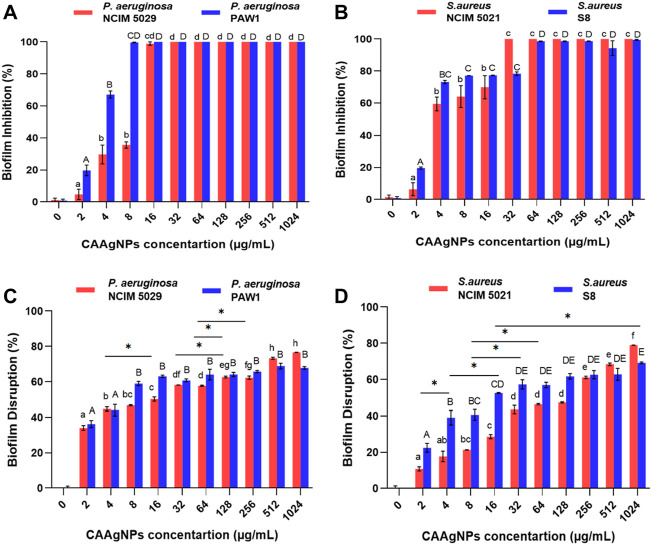
Percent biofilm inhibition of **(A)** *P. aeruginosa*, NCIM 5029 and PAW1 and **(B)** *S. aureus*, NCIM 5021 and S8 after treatment with CAAgNPs at concentrations ranging from 2 to 1,024 μg/ml. Error bars indicate standard deviation from three biological replicates. Different alphabets on top of the bars indicates differences considered statistically significant at *p* < 0.01. Percent biofilm disruption of **(C)** *P. aeruginosa*, NCIM 5029 and PAW1 and **(D)** *S. aureus*, NCIM 5021 and S8 after treatment with CAAgNPs at a concentration ranging from 2 to 1,024 μg/ml. Error bars indicate standard deviation from three biological replicates. Different alphabets on top of the bars indicates differences considered statistically significant at *p* < 0.01. Single “*” (asterisk) indicates differences considered statistically significant at *p* < 0.05.

MICs of CAAgNPs against *P. aeruginosa,* NCIM 5029 and PAW1 were 16 and 8 μg/ml respectively, whereas those for *S. aureus,* NCIM 5021 and S8 were 32 and 64 μg/ml respectively (Table 1). MBCs of CAAgNPs against *P. aeruginosa,* NCIM 5029 and PAW1 was 32 μg/ml, whereas those for *S. aureus,* NCIM 5021 and S8 was 128 μg/ml (Table 1). MICs, MBCs and MBICs of the aqueous rhizome extract of CA against all four isolates were 3 mg/ml, >3 mg/ml and 3 mg/ml respectively. MBICs of CAAgNPs against all four isolates was the same as their MICs against planktonic cells. Biofilm inhibition was observed in a dose-dependent manner for all the isolates tested (Figures 5A,B). Different letters on the bars indicate that mean values of the treatments are significantly different at *p* < 0.01 according to Tukey’s post hoc test. Error bars indicates the standard deviation from the three biological replicates.

**TABLE 1:** Antibacterial and antibiofilm activity of CAAgNPs against MDR/XDR pathogens.

Microorganisms	MICs (µg/ml)	MBCs (µg/ml)	MBICs (µg/ml)
*P. aeruginosa* NCIM 5029	16	32	16
*P. aeruginosa* PAW1	8	32	8
*S. aureus* NCIM 5021	32	128	32
*S. aureus* S8	64	128	64

Note: MICs-Minimum inhibitory concentrations; MBCs-Minimum bactericidal concentrations; MBICs-Minimum biofilm inhibitory concentrations.

### 3.3 Biofilm disruption by *Curcuma aromatica* silver nanoparticles

The treatment of 24 h old biofilms with CAAgNPs resulted in disruption of biofilms, varying between 34.08%–76.60% (*P. aeruginosa* NCIM 5029), 36.21%–67.79% (*P. aeruginosa* PAW1), 10.76%–78.94% (*S. aureus* NCIM 5021) and 22.48%–69.12% (*S. aureus* S8). Different letters on the bars indicate that mean values of treatments are significantly different at *p* < 0.01 or *p* < 0.05 (indicated with asterisk) according to Tukey’s post hoc test. Error bars indicate the standard deviation from the three biological replicates. Percent disruption in MDR clinical isolates (*P. aeruginosa* PAW1 and *S. aureus* S8) was less as compared to the antibiotic sensitive standard isolates (*P. aeruginosa* NCIM 5029 and *S. aureus* NCIM 5021). Complete eradication of biofilm was not observed in any of the four isolates tested even at CAAgNP concentrations as high as 1,024 μg/ml (Figures 5C,D).

**FIGURE 6:**
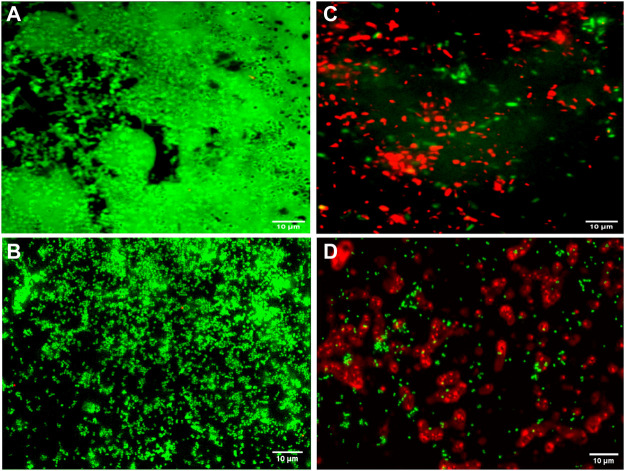
Fluorescence microscopic analysis of 24 h pre-formed biofilms under 100 × objective. Untreated pre-formed biofilms of **(A)** *P. aeruginosa* PAW1 and **(B)** *S. aureus* S8 with greater number of viable cells (green) obtained at 528 nm for SYTO9 signal. CAAgNPs treated pre-formed biofilms of **(C)** *P. aeruginosa* PAW1 and **(D)** *S. aureus* S8 with greater number of dead cells (red) obtained at 645 nm for PI signal. Few cells indicated by yellow-orange color were about to die.

**FIGURE 7:**
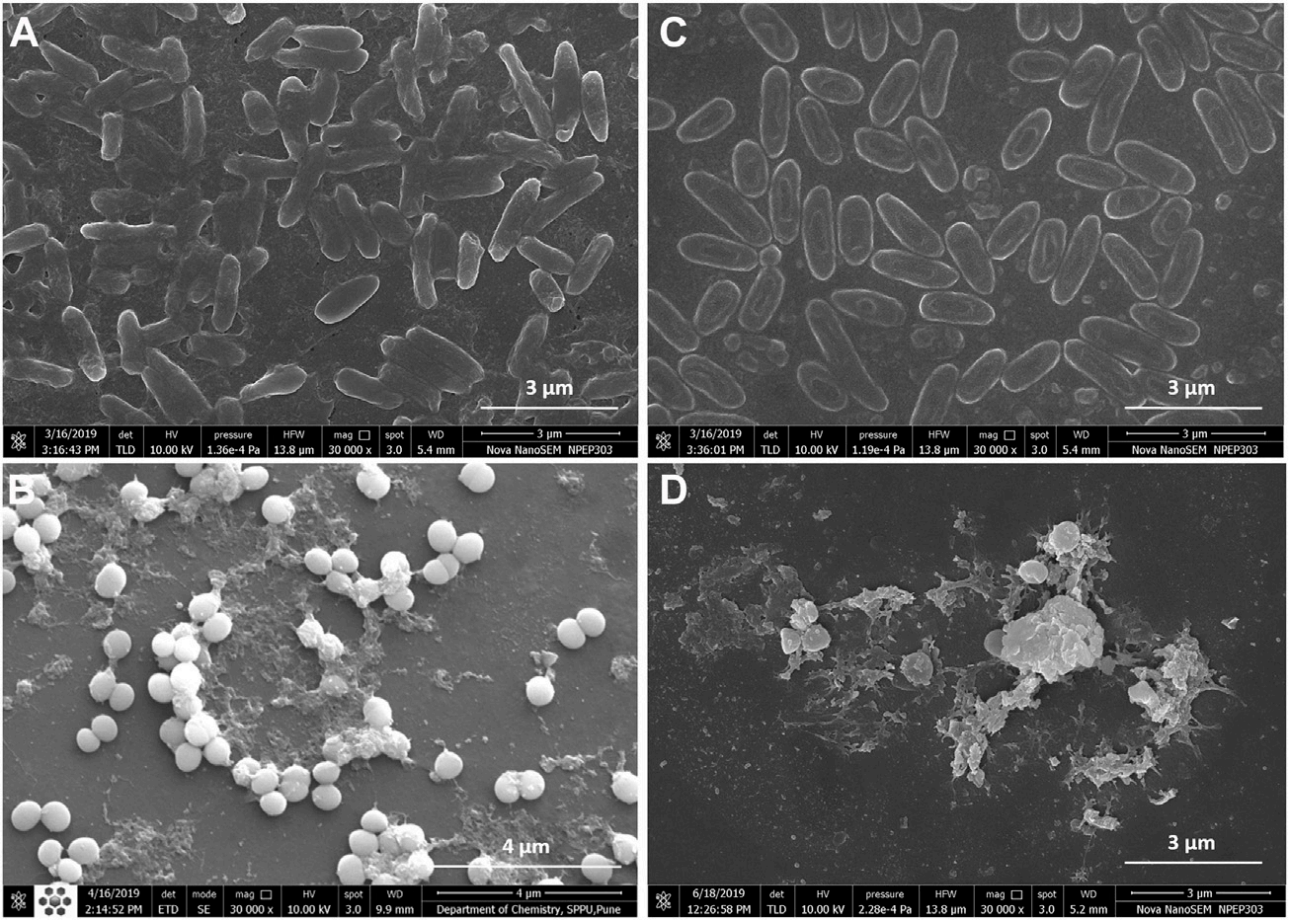
FESEM images of 24 h pre-formed biofilms at 30,000 × magnification. Untreated biofilms of **(A)** *P. aeruginosa* PAW1 and **(B)** *S. aureus* S8; CAAgNPs treated biofilms of **(C)** *P. aeruginosa* PAW1 and **(D)** *S. aureus* S8. Disruption of extracellular matrix and cell morphology was observed in treated biofilms at respective MICs.

Fluorescence microscopic images revealed that the untreated pre-formed biofilms of *P. aeruginosa* PAW1 and *S. aureus* S8 have a greater number of viable cells indicated by green color obtained at 528 nm for SYTO9 signal (Figures 6A,B). Comparatively, greater number of dead cells indicated by red color obtained at 645 nm for PI signal were observed for CAAgNPs treated pre-formed biofilms (Figures 6C,D). The cells which were about to die were indicated by yellow-orange color. FESEM analysis revealed that the untreated biofilms of *P. aeruginosa* PAW1 as well as *S. aureus* S8, were intact without any alterations in the cell morphology (Figures 7A,B). Comparatively, disruption of the cells and extracellular matrix in 24 h old biofilm of *P. aeruginosa* PAW1 and *S. aureus* S8 at their respective MICs of CAAgNps was observed (Figures 7C,D).

### 3.4 Synergistic activities of *Curcuma aromatica* silver nanoparticles in combination with antibiotics against planktonic and biofilm forms of *P. aeruginosa* PAW1

**FIGURE 8:**
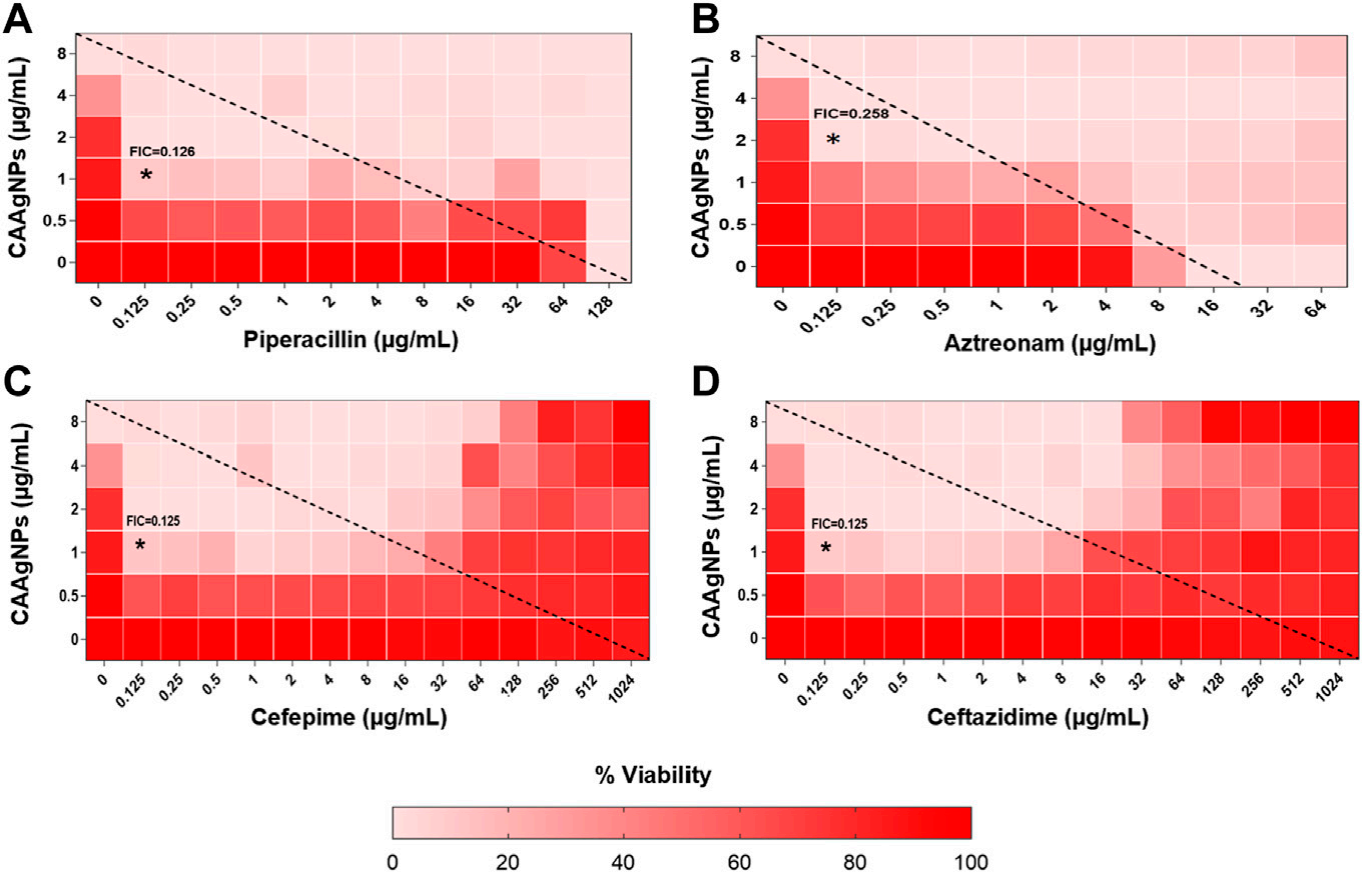
Heat plots demonstrating synergistic effect of CAAgNPs in combination with antibiotics against *P. aeruginosa* PAW1. Representation of normalized values of percent viability after treatment of CAAgNPs (0–8 μg/ml) with **(A)** Piperacillin (0–128 μg/ml), **(B)** Azetreonam (0–64 μg/ml), **(C)** Cefepime (0–1,024 μg/ml) and **(D)** ceftazidime (0–1,024 μg/ml) respectively. Untreated cells with hundred percent viability indicated by darker red boxes. Lighter red boxes indicate reduced cell viability. Dashed line passes through the MICs and “*” (asterisk) below it represents the FICi values suggesting a synergistic effect.

**FIGURE 9:**
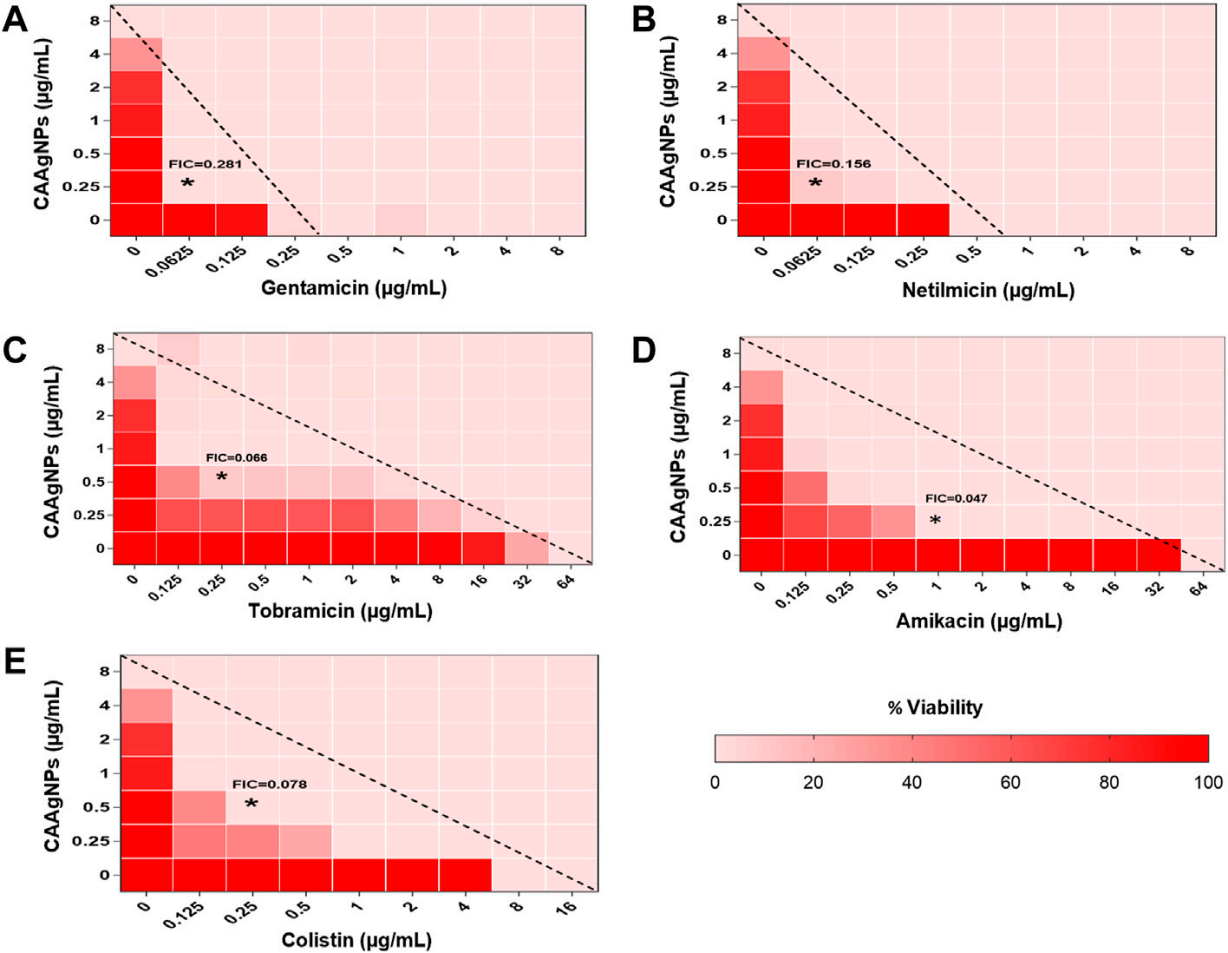
Heat plots demonstrating synergistic effect of CAAgNPs in combination with antibiotics against *P. aeruginosa* PAW1. Representation of normalized values of percent viability after treatment of CAAgNPs (0–8 μg/ml) with **(A)** Gentamicin (0–8 μg/ml), **(B)** Netilmicin (0–8 μg/ml), **(C)** Tobramycin (0–64 μg/ml), **(D)** Amikacin (0–64 μg/ml), and **(E)** Colistin (0–16 μg/ml) respectively. Untreated cells with hundred percent viability indicated by darker red boxes. Lighter red boxes indicate reduced cell viability. Dashed line passes through the MICs and “*” (asterisk) below it represents the FICi values suggesting a synergistic effect.

**FIGURE 10:**
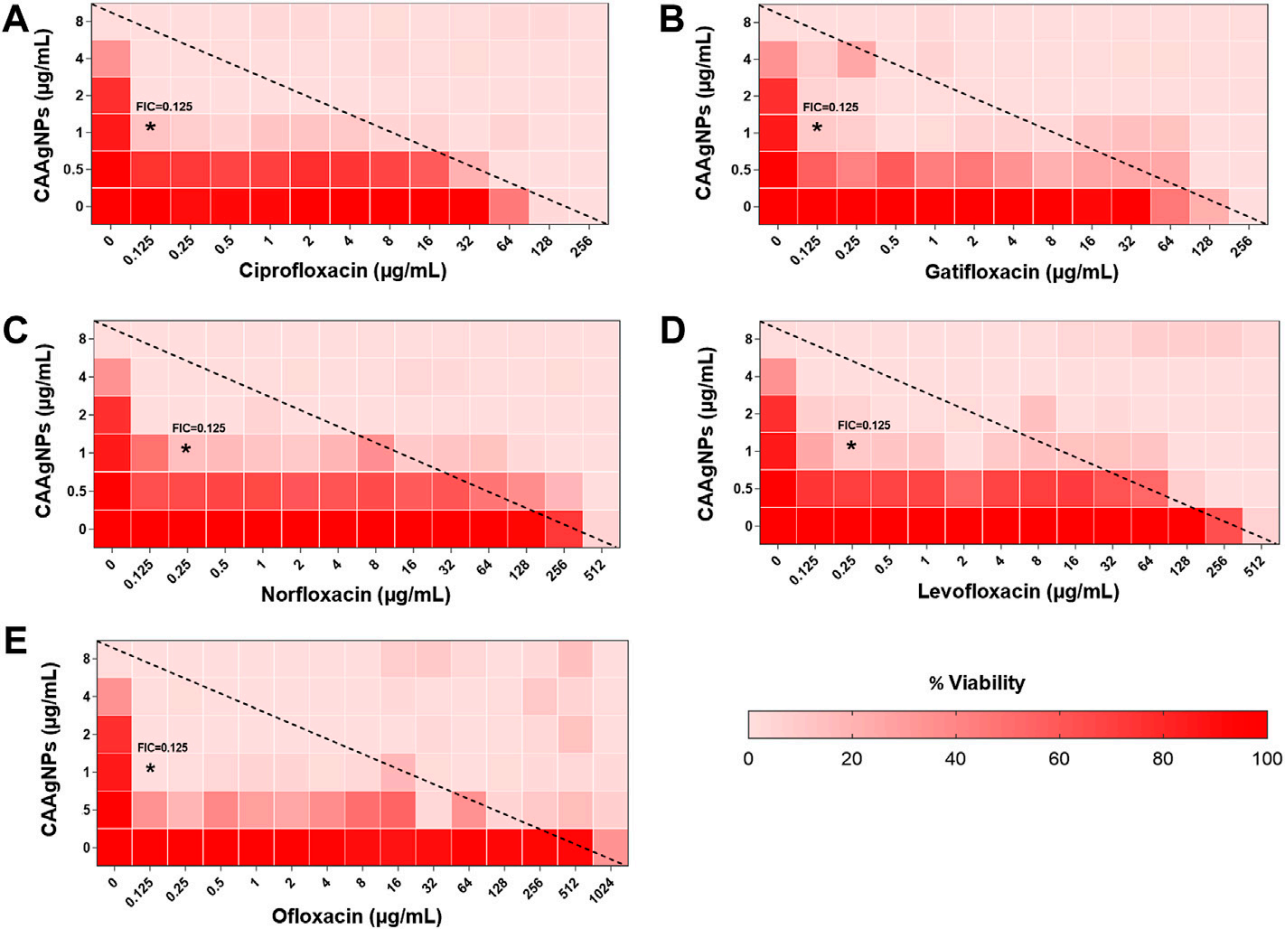
Heat plots demonstrating synergistic effect of CAAgNPs in combination with antibiotics against *P. aeruginosa* PAW1. Representation of normalized values of percent viability after treatment of CAAgNPs (0–8 μg/ml) with **(A)** Ciprofloxacin (0–256 μg/ml), **(B)** Gatifloxacin (0–256 μg/ml), **(C)** Norfloxacin (0–512 μg/ml), **(D)** Levofloxacin (0–512 μg/ml) and **(E)** Ofloxacin (0–1,024 μg/ml) respectively. Untreated cells with hundred percent viability indicated by darker red boxes. Lighter red boxes indicate reduced cell viability. Dashed line passes through the MICs and “*” (asterisk) below it represents the FICi values suggesting a synergistic effect.

**FIGURE 11:**
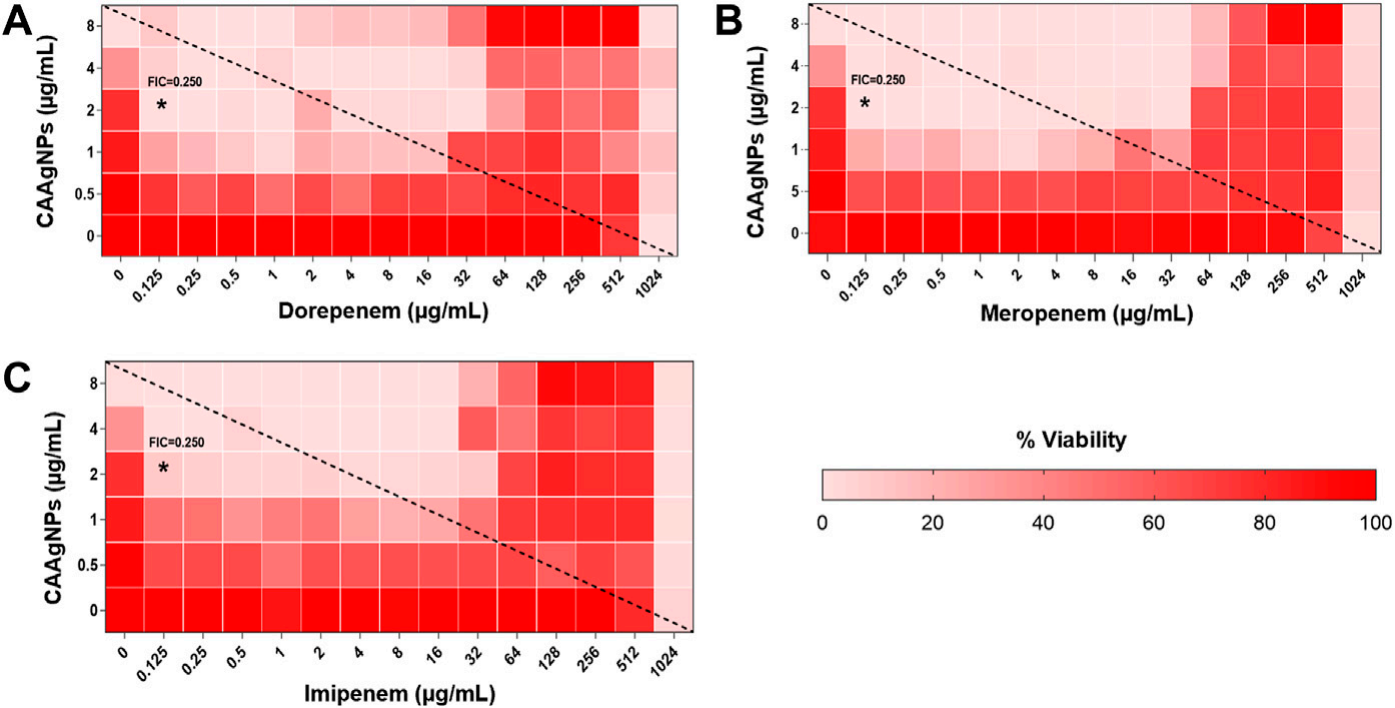
Heat plots demonstrating synergistic effect of CAAgNPs in combination with antibiotics against *P. aeruginosa* PAW1. Representation of normalized values of percent viability after treatment of CAAgNPs (0–8 μg/ml) with **(A)** dorepenem (0–1,024 μg/ml), **(B)** meropenem (0–1,024 μg/ml) and **(C)** imipenem (0–1,024 μg/ml) respectively. Untreated cells with hundred percent viability indicated by darker red boxes. Lighter red boxes indicate reduced cell viability. Dashed line passes through the MICs and “*” (asterisk) below it represents the FICi values suggesting a synergistic effect.

MICs for antibiotics against *P. aeruginosa* PAW1 were adopted from our published work (Tawre et al., 2021). Synergistic effect was observed for all combinations of CAAgNPs with antibiotics since FICi was <0.5 (Table 2). The percent viability calculated for each combination of CAAgNPs with antibiotics was represented in the form of a heat plots (Figures 8A–D; Figures 9A–E; Figures 10A–E; and Figures 11A–C). Heat plots gave a better understanding of synergistic behavior of combination of CAAgNPs with antibiotics like isobologram plots of drug-drug interaction (Tallarida, 2016). FICi value < 0.5 for each combination (indicated by an asterisk) was lying below the dashed line (passing through the respective MICs of CAAgNPs and antibiotics) which indicated synergistic interaction.

**TABLE 2:** FICi values of combination of CAAgNPs with antibiotics against planktonic and biofilm forms of *P. aeruginosa* PAW1.

Antibiotics	MICs (µg/ml)Planktonic cells			FICi	MBICs (µg/ml)Biofilm forms		FICi
	A	B	C		D	E	
Penicillins							
Piperacillin	128^R^	0.125	1	0.126	0.125	2	0.251
Monobactams							
Aztreonam	16^I^	0.125	2	0.258	4	2	0.500
Lipopeptides							
Colistin	16^I^	0.25	0.5	0.078	0.5	1	0.125
Aminoglycosides							
Gentamicin	0.25^S^	0.0625	0.25	0.281	0.0625	0.25	0.313
Tobramycin	64^R^	0.25	0.5	0.066	0.125	1	0.127
Amikacin	64^R^	1	0.25	0.047	0.25	1	0.129
Netilmicin	0.5^S^	0.0625	0.25	0.156	0.0625	0.5	0.188
Cephems							
Cefepime	>1024^R^	0.125	1	0.125	1	4	0.500
Ceftazidime	>1024^R^	0.125	1	0.125	0.5	4	0.500
Fluoroquinolones							
Ciprofloxacin	256^R^	0.125	1	0.125	0.25	2	0.251
Norfloxacin	512^R^	0.25	1	0.125	0.25	2	0.250
Levofloxacin	512^R^	0.25	1	0.125	2	2	0.254
Ofloxacin	>1024^R^	0.125	1	0.125	0.125	2	0.250
Gatifloxacin	256^R^	0.125	1	0.125	4	1	0.141
Carbapenems							
Doripenem	1024^R^	0.125	2	0.250	0.125	4	0.500
Meropenem	1024^R^	0.125	2	0.250	0.25	2	0.250
Imipenem	1024^R^	0.125	2	0.250	1	4	0.500

Note: A: MICs of antibiotics, B: lowest effective concentration of antibiotics (µg/ml) in combination with CAAgNPs; C: lowest effective concentration of CAAgNPs (µg/ml) in combination with antibiotics. S: sensitive; I: intermediate; R: resistant. FICi, represents-Synergistic: FIC≤ 0.5; Additive: 0.5 < FIC≤ 1; Indifferent: 1 < FIC< 2; Antagonistic: FIC ≥2.

Interestingly, two classes of antibiotics namely cephems (ceftazidime and cefepime) and carbapenems (meropenem, dorepenem, imipenem) in combination with CAAgNPs showed antagonistic effect at higher concentrations. Conversely, they showed synergistic effect at lower concentrations.

The FICi values for all combinations of CAAgNPs with antibiotics were ≤0.5 against *P. aeruginosa* PAW1 biofilm (Table 2) indicating a synergistic interaction.

## 4 Discussion

The emergence of antibiotic resistance has created a threat making it empirical to develop alternative treatment strategies against infections caused due to MDR pathogens. AgNPs have explicitly substantiated their role in the field of biomedicine due to their properties. Numerous medicinal plants have been employed to synthesize AgNPs and are reported for their antibacterial and antibiofilm properties against MDR pathogens (Siddiqi et al., 2018; Fahimirad et al., 2019).

Since ancient times the medicinal value of CA has been acknowledged for antibacterial and wound healing properties. The proteases from turmeric species have procoagulant and fibrinogenolytic activity. This provides a scientific basis for the traditional use of turmeric to stop bleeding and promote wound healing processes (Shivalingu et al., 2016). In the present study, it was proposed that the phytochemicals from the aqueous rhizome extract of CA may be involved in the stabilization of the CAAgNPs, and also enhancing their wound healing and antimicrobial properties.

The nature of the plant extract, its concentration, concentration of the metal salt, and other parameters such as pH, temperature and time of incubation certainly influence the yield and other characteristics of the synthesized NPs (Akhtar et al., 2013; Fahimirad et al., 2019). The phytochemicals present in the plant extract reduce the Ag^+^ ions to Ag^0^, followed by capping and stabilization of newly synthesized AgNPs (Fahimirad et al., 2019). Maximum rate of CAAgNPs synthesis was observed at 0.8 mM AgNO_3_ concentration as compared to the lower concentrations. Reduction in the rate of synthesis was seen at higher concentrations. Similarly, it has been reported that with increased concentration of AgNO_3_ it gets deposited on the surface of AgNPs by forming unclear surfaces (Fahimirad et al., 2019). There was increase in the rate of CAAgNPs synthesis with rising temperatures till 60°C with increasing incubation period till 144 h. The optimized parameters for the maximal synthesis of CAAgNPs were 0.5% (w/v) of aqueous rhizome extract of CA treated with 0.8 mM AgNO_3_ solution, incubated at 60°C for 144 h. These CAAgNPs were found to be stable, even after one year. The optimization studies certainly suggested that the experimental parameters such as concentration of AgNO_3_, temperature and reaction time have an overall impact on the synthesis of CAAgNPs. TEM technique assured the size (13 ± 5 nm) and spherical shape of the CAAgNPs. Earlier studies with AgNPs derived using CA have documented its spherical shape with few rods and triangles ranging in size of 20–40 nm (Thomas et al., 2018). Detailed characterization of CAAgNPs was not documented in the earlier report. Our study confirms the characteristics of CAAgNPs thoroughly. FESEM also confirmed the spherical shape of CAAgNPs. Further elemental mapping and EDS analysis displayed the presence of silver. XRD validated the crystalline nature of CAAgNPs. DLS revealed that the average hydrodynamic diameter of CAAgNPs was 77.88 ± 48.60 nm which was higher than the size measured using TEM. Since TEM measures the core size of CAAgNPs and DLS measures the hydrodynamic radius of CAAgNPs, which includes the coating on its surface. Earlier studies have suggested similar reports with differences in size measurement using TEM and DLS analysis (Ansar et al., 2020). Zeta potential confirmed the negative charge on CAAgNPs which keeps the particles monodispersed and prevents agglomeration. NPs with negative zeta potential values suggest there are strong repulsive forces between the NPs, that prevent the agglomeration of the NPs in solution (Majoumouo et al., 2019).

Plants and their parts contain carbohydrates, fats, proteins, nucleic acids, pigments and several types of secondary metabolites which act as reducing agents to produce nanoparticles from metal salts without producing any toxic by-product (Siddiqi et al., 2018). The phytochemical constituents of CA reported in literature include germacrone, curdione, curcumin, dehydrocurdione, zederone, curcumenol, zedoarondiol and *β*-sitosterol (Pintatum et al., 2020; Umar et al., 2020). These phytochemicals are known for various biological activities and therefore may act as reducing agents involved in synthesis of CAAgNPs and also play a role in their capping and stabilization. The FTIR absorption spectra of CA rhizome extract and CAAgNPs demonstrated shifts in peak from 1,596 cm^−1^ to 1,660 cm^−1^ which corelates with the involvement of phenolic -OH and carbonyl groups as stabilizing and reducing agent in the formation of CAAgNPs. Shift in peak from 1,214 cm^−1^ to 1,177 cm^−1^ correlates with the involvement of aromatic CO group. The presence of these functional groups confirmed that the CAAgNPs are capped with compounds from CA rhizome extract suggesting their involvement in stabilization. These results are consistent with the earlier studies reported for medicinal plants (Mohanta et al., 2020; Muniyappan et al., 2021).

The antibacterial activity of AgNPs is certainly dependent on their shape and size; the smaller the size higher is the activity (Dakal et al., 2016; Duran et al., 2016). Ag^+^ ions are released from AgNPs which then penetrate through the bacterial cell wall, rupturing it and leading to denaturation of proteins and leading to cell death (Siddiqi et al., 2018). Through the present study, we focused on antibacterial and antibiofilm activities of CAAgNPs against MDR pathogens. Lower MICs were observed against *P. aeruginosa*, NCIM 5029 (16 μg/ml) and PAW1 (8 μg/ml) as compared to *S. aureus*, NCIM 5021 (32 μg/ml) and S8 (64 μg/ml) suggesting that Gram-negative bacteria are more sensitive to CAAgNPs than Gram-positive bacteria. Comparable results were observed with AgNPs synthesized using *Cannabis sativa* in an earlier study (Singh et al., 2018b). The antibacterial studies of AgNPs against *P. aeruginosa* PAO1 have demonstrated the MIC of 12.5 μg/ml (Shah et al., 2019). In an earlier report, MICs of synthesized AgNPs against *S. aureus* were 8 μg/ml (Talank et al., 2022) and 4 μg/ml (Barabadi et al., 2021). These observations also suggests that smaller size of the AgNPs in the range 8–20 nm display lower MICs as compared to the large size AgNPs. The difference in susceptibility pattern is attributed to the thin cell wall and double cell membrane of Gram-negative bacteria through which AgNPs can penetrate (Pal et al., 2007). Contrary, penetration of AgNPs is prevented in Gram-positive bacteria due to thick cell wall with negatively charged peptidoglycan resulting in weaker antibacterial effect (Feng et al., 2000). The aqueous rhizome extract of CA displayed very high MICs, MBCs and MBICs as compared to the CAAgNPs. The antibacterial and antibiofilm activity of CAAgNPs can be attributed to the functional groups such as phenolic and aromatic from the aqueous rhizome extract of CA present on the surface of CAAgNPs. There is a possibility that these phytochemicals may be involved in the stabilization of CAAgNPs which deserve merit to be further explored. These observations are well supported with earlier studies on AgNPs derived from *Camellia sinensis,* suggesting that polyphenols of plant extract present onto the surface of AgNPs pose antimicrobial activity (Onitsuka et al., 2019). Our previous work demonstrated the potential of curcumin functionalized iron oxide nanoparticles by displaying improved inhibition of *Agrobacterium tumefaciens* when compared to bare iron oxide nanoparticles (Kitture et al., 2012). Hence, the superior antibacterial activity of CAAgNPs found in this study is well in agreement with the previous report.

One of the major reasons for acquiring drug resistance by pathogenic bacteria is biofilm formation. Elimination of biofilms becomes a major concern while treating such infections, as the pathogens do not respond to the antibiotic therapy making it imperative to develop innovative strategies. Consequently, new agents should effectively aid in inhibiting planktonic as well as biofilm forms of the pathogen (Pardesi et al., 2019). AgNPs synthesized from medicinal plants are well reported to inhibit biofilms formed by *P. aeruginosa* (Singh et al., 2018a; Singh et al., 2018b; Arya et al., 2019). The AgNPs synthesized using CA rhizome extract have also been reported to be incorporated in polymethyl methacrylate thin films which displayed antimicrobial and antibiofilm (inhibition up to 94%) activity against the cariogenic bacterium *Streptococcus mutans*. Similarly, in the present study, CAAgNPs were effective in complete biofilm inhibition of *P. aeruginosa*, NCIM 5029 and PAW1, *S. aureus,* NCIM 5021 and S8 at their respective MICs. In an earlier study, 91.02% inhibition of *S. aureus* biofilm was observed after treatment of AgNPs synthesized using *Z. multiflora* (Barabadi et al., 2021). In another study, treatment with three types of AgNPs displayed >99% of biofilm inhibition at 100, 50 and 60 μg/ml concentrations for *P. aeruginosa* and at 90, 60 and 60 μg/ml concentrations for *S. aureus* (Mohanta et al., 2020). Although biofilm disruption assay demonstrated around 50% reduction in the biofilm mass at the respective MICs, however, >1,024 μg/ml of concentration was required for the complete disruption of biofilm. Live/Dead staining of CAAgNPs treated pre-formed biofilms of *P. aeruginosa* PAW1 and *S. aureus* S8 revealed that very few cells were viable (green) and greater number of the cells were dead (dead). Disruption of pre-formed biofilms matrix of *P. aeruginosa* PAW1 and *S. aureus* S8 observed through FESEM analysis also demonstrated the antibiofilm potential of CAAgNPs. Reduction in the cell numbers and disruption of the cell surface morphology as compared to untreated control was observed. Comparable results were observed in earlier studies after treatment of pre-formed biofilms of *P. aeruginosa* and *S. aureus* with AgNPs (Singh et al., 2018b; Singh et al., 2019a; Singh et al., 2019b). These findings suggest a possibility that Ag^+^ ions are released from CAAgNPs that adhere to the cell membrane, internalize through cell membrane, damage the cell components, and consequently leads to the cell death.

Repeated use of antibiotics has developed resistance towards the existing antibiotics and currently no new antibiotics are in the pipeline (Mulani et al., 2019). Similarly, it is conveyed in an earlier study that repeated exposure to AgNPs may develop resistance in the organisms (Panacek et al., 2018). Consequently, alternate strategies such as combination therapies involving antibiotics with AgNPs to treat MDR pathogens should come into play. Such combination therapy will reduce the dosage of antibiotic and AgNPs well below their MICs which otherwise required a high amount to exhibit the inhibitory effects on MDR pathogens. Reduced dosage will minimize any toxic effects on the host cells.

Representation of percent viability of *P. aeruginosa* PAW1 treated with CAAgNPs in combination with antibiotics using heat plots gives a better understanding of synergism. In the present study, the combination of CAAgNPs with antibiotics from class, penicillins, monobactams, aminoglycosides and fluoroquinolones suggested synergistic effects (FICi <0.5). Similar results were obtained in another study where AgNPs were used in combination with aztreonam and tobramycin against *P. aeruginosa* PAO1 biofilms. Smaller size AgNPs (10 and 20 nm) were found to be more effective at lower concentrations as compared to larger size AgNPs (40, 60 and 100 nm). The study also suggests that the antimicrobial activity of AgNPs can be affected by the factors such as strain dependent differences, source of AgNPs synthesis and surface modifications. (Habash et al., 2014; Habash et al., 2017). The efficacy of polymyxin B in combination with AgNPs was enhanced as compared to polymyxin B alone (Salman et al., 2019). AgNPs synthesized using *D. bulbifera* tuber extract have displayed synergistic effect in combination with antibiotics against *P. aeruginosa* (Ghosh et al., 2012). Earlier report has demonstrated non-specific synergistic activity of antibiotics with AgNPs against *P. aeruginosa* and *S. aureus* (Panacek et al., 2015). Interestingly, synergistic effect was also observed with two classes of antibiotics namely cephems (ceftazidime and cefepime) and carbapenems (meropenem, imipenem or dorepenem) at lower concentrations when in combination with CAAgNPs, however, an antagonistic effect was observed at higher concentrations.

Earlier reports have suggested the role of AgNPs in altering the cell membrane integrity, leading to increase in the cell permeability thereby allowing the entry of antibiotics inside the cell (Vazquez-Munoz et al., 2019). Further studies to understand the mechanistic action of CAAgNPs therefore needs to be investigated. Antagonistic interactions indicate a possibility that high antibiotic concentrations (of cephems and carbapenem class) just below the MIC values hinder the CAAgNPs activity. This observation also needs to be further investigated to understand the mechanism that leads to antagonistic effects.

Complete inhibition of biofilm was observed with CAAgNPs in combination with antibiotics which occurred at concentrations much below their individual MICs and displayed synergistic effect. In earlier reports, the synergistic effect of AgNPs with polymyxin B has been reported against *P. aeruginosa* biofilm. This further affirms the role of CAAgNPs as an effective antibacterial and antibiofilm agent. FICi of CAAgNPs in combination with antibiotics obtained using MBICs indicated their synergistic effect on biofilm forms of *P. aeruginosa* PAW1.

The use of AgNPs as antibiofilm coatings in surgical implants, antimicrobial agents in topical applications, or as formulations in wound dressings has shown promising results in animal models (Mulani et al., 2019). However, there is a thin line in between the *in vitro* studies being performed and execution of these findings into the clinical trials. There is a noteworthy advancement where AgNPs were incorporated in topical gel for antimicrobial activity in a phase I trial (Clinical Trial Registration: NCT03752424). More such agents should be clinically tested in terms of their efficacy and safety to be made commercially available in the market. Therefore, before employing AgNPs in medicine, their biocompatibility becomes a crucial factor (Qing et al., 2018). Low toxicity of AgNPs derived using *Lysiloma acapulcensis* towards human peripheral blood lymphocytes has been documented (Garibo et al., 2020). In an earlier report, the effect of AgNPs on PBMCs was tested considering the possibility that AgNPs may penetrate the skin and enter the bloodstream. AgNPs were found to be nontoxic towards PBMCs (Banasiuk et al., 2016). In our earlier study, CAAgNPs were tested for their toxic effects on PBMC’s (Nadhe et al., 2020) and showed IC_50_ > 200 μg/ml which is much higher than the MICs against MDR pathogens reported in the present study. These findings suggests that CAAgNPs have the potential to be used for therapeutic applications against MDR pathogens as they exhibit low toxic effects on PBMCs. *In vivo* safety and efficacy studies of CAAgNPs concerning mice models becomes imperative before the execution of CAAgNPs for further biomedical applications. Forthcoming studies focusing on mechanistic action of CAAgNPs such as inhibition of efflux pump activity, membrane permeabilization and change in membrane potential of bacterial cells needs to be investigated.

## 5 Conclusion

Overall, CAAgNPs are promising candidates to be used as antibacterial as well as antibiofilm agents against MDR pathogens (singly or in combination with antibiotics). Combination studies revealed that the required concentrations of antibiotics and CAAgNPs can be further lowered, thereby, reducing the toxic effects caused, if any. The low toxicity of CAAgNPs observed against PBMCs suggests their potential use in biomedical applications. We recommend possible uses of CAAgNPs in the preparation of wound dressings, gels or ointments, and coating of medical devices, catheters, and dental and orthopedic implants.

## Data Availability

The original contributions presented in the study are included in the article, further inquiries can be directed to the corresponding author.

## References

[B1] Akhtar M. S. Panwar J. Yun Y. S. (2013). Biogenic synthesis of metallic nanoparticles by plant extracts. ACS Sustain. Chem. Eng. 1, 591–602. 10.1021/SC300118U

[B2] Altinsoy B. D. Şeker Karatoprak G. Ocsoy I. (2019). Extracellular directed ag NPs formation and investigation of their antimicrobial and cytotoxic properties. Saudi Pharm. J. 27, 9–16. 10.1016/J.JSPS.2018.07.013 30627047 PMC6323131

[B3] Ansar S. Tabassum H. Aladwan N. S. M. Naiman Ali M. Almaarik B. AlMahrouqi S. (2020). Eco friendly silver nanoparticles synthesis by *Brassica oleracea* and its antibacterial, anticancer and antioxidant properties. Sci. Rep. 10, 18564–18612. 10.1038/s41598-020-74371-8 33122798 PMC7596502

[B4] Arya G. Kumari R. M. Sharma N. Gupta N. Kumar A. Chatterjee S. (2019). Catalytic, antibacterial and antibiofilm efficacy of biosynthesised silver nanoparticles using *Prosopis juliflora* leaf extract along with their wound healing potential. J. Photochem. Photobiol. B Biol. 190, 50–58. 10.1016/j.jphotobiol.2018.11.005 30472614

[B5] Banasiuk R. Frackowiak J. E. Krychowiak M. Matuszewska M. Kawiak A. Ziabka M. (2016). Synthesis of antimicrobial silver nanoparticles through a photomediated reaction in an aqueous environment. Int. J. Nanomedicine 11, 315–324. 10.2147/IJN.S93611 26855570 PMC4725629

[B6] Barabadi H. Mojab F. Vahidi H. Marashi B. Talank N. Hosseini O. (2021). Green synthesis, characterization, antibacterial and biofilm inhibitory activity of silver nanoparticles compared to commercial silver nanoparticles. Inorg. Chem. Commun. 129, 108647. 10.1016/J.INOCHE.2021.108647

[B7] Berenbaum M. C. (1978). A method for testing for synergy with any number of agents. J. Infect. Dis. 137, 122–130. 10.1093/INFDIS/137.2.122 627734

[B8] Cheon J. Y. Kim S. J. Rhee Y. H. Kwon O. H. Park W. H. (2019). Shape-dependent antimicrobial activities of silver nanoparticles. Int. J. Nanomedicine 14, 2773–2780. 10.2147/IJN.S196472 31118610 PMC6499446

[B9] Dakal T. C. Kumar A. Majumdar R. S. Yadav V. (2016). Mechanistic basis of antimicrobial actions of silver nanoparticles. Front. Microbiol. 7, 1831. 10.3389/fmicb.2016.01831 27899918 PMC5110546

[B10] Duran N. Duran M. de Jesus M. B. Seabra A. B. Favaro W. J. Nakazato G. (2016). Silver nanoparticles: A new view on mechanistic aspects on antimicrobial activity. Nanomedicine Nanotechnol. Biol. Med. 12, 789–799. 10.1016/J.NANO.2015.11.016 26724539

[B11] Fahimirad S. Ajalloueian F. Ghorbanpour M. (2019). Synthesis and therapeutic potential of silver nanomaterials derived from plant extracts. Ecotoxicol. Environ. Saf. 168, 260–278. 10.1016/j.ecoenv.2018.10.017 30388544

[B12] Feng Q. L. Wu J. Chen G. Q. Cui F. Z. Kim T. N. Kim J. O. (2000). A mechanistic study of the antibacterial effect of silver ions on *Escherichia coli* and *Staphylococcus aureus* . J. Biomed. Mat. Res. 52, 662–668. 10.1002/1097-4636(20001215)52:4<662::AID-JBM10>3.0.CO;2-3 11033548

[B14] Gafur A. Sukamdani G. Y. Kristi N. Maruf A. Xu J. Chen X. (2020). From bulk to nano-delivery of essential phytochemicals: Recent progress and strategies for antibacterial resistance. J. Mat. Chem. B 8, 9825–9835. 10.1039/D0TB01671C 33000844

[B15] Gaidhani S. V. Raskar A. V. Poddar S. Gosavi S. Sahu P. K. Pardesi K. R. (2014). Time dependent enhanced resistance against antibiotics & metal salts by planktonic & biofilm form of *Acinetobacter haemolyticus* MMC 8 clinical isolate. Indian J. Med. Res. 140, 665–671. Available: /pmc/articles/PMC4311322/?report = abstract 25579150 PMC4311322

[B16] Garibo D. Borbon-Nunez H. A. de Leon J. N. D. Garcia Mendoza E. Estrada I. Toledano-Magana Y. (2020). Green synthesis of silver nanoparticles using *Lysiloma acapulcensis* exhibit high-antimicrobial activity. Sci. Rep. 10, 12805–12811. 10.1038/s41598-020-69606-7 32732959 PMC7393152

[B17] Ghosh S. Patil S. Ahire M. Kitture R. Kale S. Pardesi K. (2012). Synthesis of silver nanoparticles using *Dioscorea bulbifera* tuber extract and evaluation of its synergistic potential in combination with antimicrobial agents. Int. J. Nanomedicine 483, 483–496. 10.2147/ijn.s24793 PMC3273981 22334779

[B18] Ghosh S. Bloch K. Webster T. J. (2021). Functionalized biogenic nanoparticles and their applications. Nanobiotechnology 2021, 303–322. 10.1016/B978-0-12-822878-4.00019-5

[B19] Habash M. B. Goodyear M. C. Park A. J. Surette M. D. Vis E. C. Harris R. J. (2017). Potentiation of tobramycin by silver nanoparticles against *Pseudomonas aeruginosa* biofilms. Antimicrob. Agents Chemother. 61, e00415–e00417. 10.1128/AAC.00415-17 28848007 PMC5655055

[B20] Habash M. B. Park A. J. Vis E. C. Harris R. J. Khursigara C. M. (2014). Synergy of silver nanoparticles and aztreonam against *Pseudomonas aeruginosa* PAO1 Biofilms. Antimicrob. Agents Chemother. 58, 5818–5830. 10.1128/AAC.03170-14 25049240 PMC4187931

[B21] Javed B. Ikram M. Farooq F. Sultana T. Mashwani Z.-U.-R. Naveed I. R. (2021). Biogenesis of silver nanoparticles to treat cancer, diabetes, and microbial infections: A mechanistic overview. Appl. Microbiol. Biotechnol. 105, 2261–2275. 10.1007/s00253-021-11171-8 33591386

[B22] Kamble E. Pardesi K. (2020). Antibiotic tolerance in biofilm and stationary-phase planktonic cells of *Staphylococcus aureus* . Microb. Drug Resist. 00, 3–12. 10.1089/mdr.2019.0425 32013708

[B23] Kitture R. Ghosh S. Kulkarni P. Liu X. L. Maity D. Patil S. I. (2012). Fe 3O 4-citrate-curcumin: Promising conjugates for superoxide scavenging, tumor suppression and cancer hyperthermia. J. Appl. Phys. 111, 064702. 10.1063/1.3696001

[B24] Koo H. Allan R. N. Howlin R. P. Stoodley P. Hall-Stoodley L. (2017). Targeting microbial biofilms: Current and prospective therapeutic strategies. Nat. Rev. Microbiol. 15, 740–755. 10.1038/nrmicro.2017.99 28944770 PMC5685531

[B25] Macovei I. Luca S. V. Skalicka-Woźniak K. Sacarescu L. Pascariu P. Ghilan A. (2022). Phyto-functionalized silver nanoparticles derived from conifer bark extracts and evaluation of their antimicrobial and cytogenotoxic effects. Molecules 27, 217. 10.3390/MOLECULES27010217 PMC8746316 35011449

[B26] Majoumouo M. S. Sibuyi N. R. S. Tincho M. B. Mbekou M. Boyom F. F. Meyer M. (2019). Enhanced anti-bacterial activity of biogenic silver nanoparticles synthesized from *Terminalia mantaly* extracts. Int. J. Nanomedicine 14, 9031–9046. 10.2147/IJN.S223447 31819417 PMC6875292

[B27] Manosalva N. Tortella G. Cristina Diez M. Schalchli H. Seabra A. B. Durán N. (2019). Green synthesis of silver nanoparticles: Effect of synthesis reaction parameters on antimicrobial activity. World J. Microbiol. Biotechnol. 35, 88. 10.1007/s11274-019-2664-3 31134435

[B28] Mohanta Y. K. Biswas K. Jena S. K. Hashem A. AbdAllah E. F. Mohanta T. K. (2020). Anti-biofilm and antibacterial activities of silver nanoparticles synthesized by the reducing activity of phytoconstituents present in the Indian medicinal plants. Front. Microbiol. 11, 1143. 10.3389/fmicb.2020.01143 32655511 PMC7324531

[B29] Mostafavi E. Zarepour A. Barabadi H. Zarrabi A. Truong L. B. Medina-Cruz D. (2022). Antineoplastic activity of biogenic silver and gold nanoparticles to combat leukemia: Beginning a new era in cancer theragnostic. Biotechnol. Rep. (Amst). 34, e00714. 10.1016/J.BTRE.2022.E00714 35686001 PMC9171450

[B30] Mulani M. S. Kamble E. E. Kumkar S. N. Tawre M. S. Pardesi K. R. (2019). Emerging strategies to combat ESKAPE pathogens in the era of antimicrobial resistance: A review. Front. Microbiol. 10, 539. 10.3389/fmicb.2019.00539 30988669 PMC6452778

[B31] Muniyappan N. Pandeeswaran M. Amalraj A. (2021). Green synthesis of gold nanoparticles using *Curcuma pseudomontana* isolated curcumin: Its characterization, antimicrobial, antioxidant and anti- inflammatory activities. Environ. Chem. Ecotoxicol. 3, 117–124. 10.1016/J.ENCECO.2021.01.002

[B32] Nadhe S. B. Tawre M. S. Agrawal S. Chopade B. A. Sarkar D. Pardesi K. (2020). Anticancer potential of AgNPs synthesized using *Acinetobacter* sp. and *Curcuma aromatica* against HeLa cell lines: A comparative study. J. Trace Elem. Med. Biol. 62, 126630. 10.1016/J.JTEMB.2020.126630 32738757

[B33] Onitsuka S. Hamada T. Okamura H. (2019). Preparation of antimicrobial gold and silver nanoparticles from tea leaf extracts. Colloids and Surfaces B: Biointerfaces 173, 242–248. 10.1016/j.colsurfb.2018.09.055 30300830

[B34] Pal S. Tak Y. K. Song J. M. (2007). Does the antibacterial activity of silver nanoparticles depend on the shape of the nanoparticle? A study of the Gram-negative bacterium *Escherichia coli* . Appl. Environ. Microbiol. 73, 1712–1720. 10.1128/AEM.02218-06 17261510 PMC1828795

[B35] Panacek A. Kvitek L. Smekalova M. Vecerova R. Kolar M. Roderova M. (2018). Bacterial resistance to silver nanoparticles and how to overcome it. Nat. Nanotechnol. 13, 65–71. 10.1038/s41565-017-0013-y 29203912

[B36] Panacek A. Smekalova M. Kilianova M. Prucek R. Bogdanova K. Vecerova R. (2015). Strong and nonspecific synergistic antibacterial efficiency of antibiotics combined with silver nanoparticles at very low concentrations showing no cytotoxic effect. Molecules 21, 26. 10.3390/molecules21010026 26729075 PMC6273824

[B37] Pardesi K. R. Pable A. A. Bhagat D. D. Satpute S. K. (2019). “Applications of metal nanoparticles to combat biofilm forming ESKAPE pathogens,” in Recent advances in Biotechnology (Nova Science Publisher).

[B38] Parit S. B. Karade V. C. Patil R. B. Pawar N. V. Dhavale R. P. Tawre M. (2020). Bioinspired synthesis of multifunctional silver nanoparticles for enhanced antimicrobial and catalytic applications with tailored SPR properties. Mat. Today Chem. 17, 100285. 10.1016/J.MTCHEM.2020.100285

[B39] Pintatum A. Maneerat W. Logie E. Tuenter E. Sakavitsi M. E. Pieters L. (2020). *In vitro* anti-inflammatory, anti-oxidant, and cytotoxic activities of four *Curcuma* species and the isolation of compounds from *Curcuma aromatica* rhizome. Biomolecules 10, 799. 10.3390/BIOM10050799 32455782 PMC7277146

[B40] Qing Y. Cheng L. Li R. Liu G. Zhang Y. Tang X. (2018). Potential antibacterial mechanism of silver nanoparticles and the optimization of orthopedic implants by advanced modification technologies. Int. J. Nanomedicine 13, 3311–3327. 10.2147/IJN.S165125 29892194 PMC5993028

[B41] Rai M. Yadav A. Gade A. (2009). Silver nanoparticles as a new generation of antimicrobials. Biotechnol. Adv. 27, 76–83. 10.1016/j.biotechadv.2008.09.002 18854209

[B42] Rashid S. Azeem M. Khan S. A. Shah M. M. Ahmad R. (2019). Characterization and synergistic antibacterial potential of green synthesized silver nanoparticles using aqueous root extracts of important medicinal plants of Pakistan. Colloids and Surfaces B: Biointerfaces 179, 317–325. 10.1016/j.colsurfb.2019.04.016 30981067

[B43] Revathi S. Malathy N. S. (2013). Antibacterial activity of rhizome of *Curcuma aromatica* and partial purification of active compounds. Indian J. Pharm. Sci. 75, 732–735. 24591751 PMC3928740

[B44] Salman M. Rizwana R. Khan H. Munir I. Hamayun M. Iqbal A. (2019). Synergistic effect of silver nanoparticles and polymyxin B against biofilm produced by *Pseudomonas aeruginosa* isolates of pus samples *in vitro* . Artif. Cells Nanomed. Biotechnol. 47, 2465–2472. 10.1080/21691401.2019.1626864 31187657

[B45] Shah S. Gaikwad S. Nagar S. Kulshrestha S. Vaidya V. Nawani N. (2019). Biofilm inhibition and anti-quorum sensing activity of phytosynthesized silver nanoparticles against the nosocomial pathogen *Pseudomonas aeruginosa* . Biofouling 35, 34–49. 10.1080/08927014.2018.1563686 30727758

[B46] Shivalingu B. R. Vivek H. K. Priya B. S. Soujanya K. N. Nanjunda Swamy S. (2016). Purification and characterization of novel fibrin(ogen)olytic protease from *Curcuma aromatica* Salisb. Role in hemostasis. Phytomedicine 23, 1691–1698. 10.1016/j.phymed.2016.09.007 27823634

[B47] Siddiqi K. S. Husen A. Rao R. A. K. (2018). A review on biosynthesis of silver nanoparticles and their biocidal properties. J. Nanobiotechnology 16, 14–28. 10.1186/s12951-018-0334-5 29452593 PMC5815253

[B48] Singh B. P. Ghosh S. Chauhan A. (2021). Development, dynamics and control of antimicrobial-resistant bacterial biofilms: A review. Environ. Chem. Lett. 19, 1983–1993. 10.1007/s10311-020-01169-5

[B49] Singh N. Paknikar K. M. Rajwade J. (2019a). RNA-sequencing reveals a multitude of effects of silver nanoparticles on *Pseudomonas aeruginosa* biofilms. Environ. Sci. Nano 6, 1812–1828. 10.1039/C8EN01286E

[B50] Singh N. Rajwade J. Paknikar K. M. (2019b). Transcriptome analysis of silver nanoparticles treated *Staphylococcus aureus* reveals potential targets for biofilm inhibition. Colloids and Surfaces B: Biointerfaces 175, 487–497. 10.1016/J.COLSURFB.2018.12.032 30572157

[B51] Singh P. Pandit S. Beshay M. Mokkapati V. R. S. S. Garnaes J. Olsson M. E. (2018a). Anti-biofilm effects of gold and silver nanoparticles synthesized by the *Rhodiola rosea* rhizome extracts. Artif. Cells Nanomed. Biotechnol. 46, S886–S899. 10.1080/21691401.2018.1518909 30422688

[B52] Singh P. Pandit S. Garnæs J. Tunjic S. Mokkapati V. R. S. S. Sultan A. (2018b). Green synthesis of gold and silver nanoparticles from *Cannabis sativa* (Industrial hemp) and their capacity for biofilm inhibition. Int. J. Nanomedicine 13, 3571–3591. 10.2147/IJN.S157958 29950836 PMC6016601

[B53] Swilam N. Nematallah K. A. (2020). Polyphenols profile of pomegranate leaves and their role in green synthesis of silver nanoparticles. Sci. Rep. 10, 14851–14911. 10.1038/s41598-020-71847-5 32908245 PMC7481211

[B54] Talank N. Morad H. Barabadi H. Mojab F. Amidi S. Kobarfard F. (2022). Bioengineering of green-synthesized silver nanoparticles: *In vitro* physicochemical, antibacterial, biofilm inhibitory, anticoagulant, and antioxidant performance. Talanta 243, 123374. 10.1016/J.TALANTA.2022.123374 35298927

[B55] Tallarida R. J. (2016). Drug combinations: Tests and analysis with isoboles. Curr. Protoc. Pharmacol. 72, 9191. 10.1002/0471141755.PH0919S72 PMC4839184 26995550

[B56] Tawre M. S. Kamble E. E. Kumkar S. N. Mulani M. S. Pardesi K. R. (2021). Antibiofilm and antipersister activity of acetic acid against extensively drug resistant *Pseudomonas aeruginosa* PAW1. PLoS One 16, e0246020. 10.1371/JOURNAL.PONE.0246020 33529248 PMC7853517

[B57] Thomas R. Snigdha S. Bhavitha K. B. Babu S. Ajith A. Radhakrishnan E. K. (2018). Biofabricated silver nanoparticles incorporated polymethyl methacrylate as a dental adhesive material with antibacterial and antibiofilm activity against *Streptococcus mutans* . 3 Biotech 38, 404. 10.1007/s13205-018-1420-y PMC6131137 30221117

[B58] Tripathi N. Goshisht M. K. (2022). Recent advances and mechanistic insights into antibacterial activity, antibiofilm activity, and cytotoxicity of silver nanoparticles. ACS Appl. bio Mat. 5, 1391–1463. 10.1021/ACSABM.2C00014 35358388

[B59] Umar N. M. Parumasivam T. Aminu N. Toh S.-M. (2020). Phytochemical and pharmacological properties of *Curcuma aromatica* Salisb (wild turmeric). J. Appl. Pharm. Sci. 10, 180–194. 10.7324/JAPS.2020.1010018

[B60] Vazquez-Munoz R. Meza-Villezcas A. Fournier P. G. J. Soria-Castro E. Juarez-Moreno K. Gallego-Hernandez A. L. (2019). Enhancement of antibiotics antimicrobial activity due to the silver nanoparticles impact on the cell membrane. PLoS One 14, e0224904. 10.1371/JOURNAL.PONE.0224904 31703098 PMC6839893

[B61] Woo K. J. Hye C. K. Ki W. K. Shin S. So H. K. Yong H. P. (2008). Antibacterial activity and mechanism of action of the silver ion in *Staphylococcus aureus* and *Escherichia coli* . Appl. Environ. Microbiol. 74, 2171–2178. 10.1128/AEM.02001-07 18245232 PMC2292600

